# PIN1 Provides Dynamic Control of MYC in Response to Extrinsic Signals

**DOI:** 10.3389/fcell.2020.00224

**Published:** 2020-04-02

**Authors:** Gabriel M. Cohn, Daniel F. Liefwalker, Ellen M. Langer, Rosalie C. Sears

**Affiliations:** ^1^Department of Molecular and Medical Genetics, School of Medicine, Oregon Health and Science University, Portland, OR, United States; ^2^Knight Cancer Institute, Oregon Health and Science University, Portland, OR, United States; ^3^Brenden-Colson Center for Pancreatic Care, Oregon Health and Science University, Portland, OR, United States

**Keywords:** Pin1, c-Myc, nuclear pore complex, phosphorylation, isomerization

## Abstract

PIN1 is a phosphorylation-directed member of the peptidyl-prolyl *cis*/*trans* isomerase (PPIase) family that facilitates conformational changes in phosphorylated targets such as c-MYC (MYC). Following signaling events that mediate phosphorylation of MYC at Serine 62, PIN1 establishes structurally distinct pools of MYC through its *trans-cis* and *cis-trans* isomerization activity at Proline 63. Through these isomerization steps, PIN1 functionally regulates MYC’s stability, the molecular timing of its DNA binding and transcriptional activity, and its subnuclear localization. Recently, our group showed that Serine 62 phosphorylated MYC can associate with the inner basket of the nuclear pore (NP) in a PIN1-dependent manner. The poised euchromatin at the NP basket enables rapid cellular response to environmental signals and cell stress, and PIN1-mediated trafficking of MYC calibrates this response. In this perspective, we describe the molecular aspects of PIN1 target recognition and PIN1’s function in the context of its temporal and spatial regulation of MYC.

## Introduction

Proline isomerization of cellular proteins provides post-translational control of target protein structure, and therefore function, within the cell. Proline residues within peptides can exist in two distinct energetically stable states, *cis* or *trans*. While proline residues exhibit an intrinsic ability to isomerize, this process occurs on a very slow biomolecular timescale as a result of the high-energy barrier associated with this conformational change. This high-energy barrier isolates the *cis* and *trans* protein states, and rapidly switching between these two conformational states requires a catalyst. The evolutionarily conserved peptidyl-prolyl *cis*/*trans* isomerases (PPIases) catalyze this conformational change and are required to drive isomerization in a timeframe relevant to dynamic signaling cascades within the cell (Lu et al., 2007; Chen Y. et al., 2018). By functioning as molecular switches to toggle targets between their *cis* and *trans* conformations, these enzymes can affect target protein stability, localization, activity, and protein–protein interactions (Göthel and Marahiel, 1999; Lu et al., 2007; Takahashi et al., 2008).

The PPIase, NIMA-interacting 1 (PIN1) is the only known PPIase that specifically recognizes phosphorylated serine or threonine residues that immediately precede a proline (pSer/pThr-Pro). This pSer/pThr-Pro motif accounts for over 25% of all phosphorylation sites identified in a global phosphorylation study (Ubersax and Ferrell, 2007). The proline-directed kinases that target these sites are central to extracellular stimuli responses (Pearson et al., 2001) and cell cycle progression (Morgan, 1997; Cheng and Tse, 2018). The selectivity of PIN1 for phosphorylated proteins provides it with the potential to modify and functionally regulate a variety of targets involved in these phospho-signaling cascades. Indeed, PIN1 has been shown to target important cell cycle phospho-proteins such as Cyclin D1 (Liou et al., 2002) as well as proteins in the NF-κB, WNT, and AKT pathways, where extrinsic signals result in phosphorylation-regulated cascades that ultimately alter gene transcription to affect cell phenotype (Ryo et al., 2001, 2003; Liao et al., 2009). Despite PIN1’s involvement in critical signaling pathways, PIN1 null mice are viable. The major phenotype of mice lacking PIN1 is a defect in cellular proliferation that contributes to stunted body size and infertility (Fujimori et al., 1999; Liou et al., 2002). Consistent with this, mouse embryonic fibroblasts (MEFs) from PIN1 knockout mice, that exhibit similar proliferation relative to wildtype (WT) MEFs during asynchronous growth in culture, display significantly delayed proliferation relative to WT MEFs when stimulated with mitogens after being starved to G_0_ arrest (Fujimori et al., 1999; Su et al., 2018). This result supports an important role for PIN1 in dynamic signaling pathways to elicit an efficient response to extracellular stimuli.

Loss of PIN1 also renders cells resistant to transformation and, strikingly, PIN1 knockout mice have delayed tumor formation when crossed with tumor-driving mutants of HER2 or RAS (Ryo et al., 2002; Wulf et al., 2004). Phospho-signaling is increased in cancer, often in a cell-intrinsic manner by oncogenic mutations in signaling pathways (e.g., RAS or HER2), but also through cell-extrinsic signals from the tumor microenvironment (e.g., TGFβ or FGF). These conditions lead to an abundance of proline-directed kinases driving oncogenic signaling cascades that control tumorigenic phenotypes (Gross et al., 2015). PIN1 regulates a large number of these cancer-related targets from extracellular receptors such as NOTCH1 (Rustighi et al., 2009) or HER2 (Lam et al., 2008), to intracellular effector proteins like RAF1 (Dougherty et al., 2005) or FAK (Zheng et al., 2009), and ultimately to transcription factors such as c-MYC (Farrell et al., 2013), β-catenin (Ryo et al., 2001), or NF-κB (Ryo et al., 2003). The overexpression of PIN1 is common in many types of cancer and is correlated with poor outcomes (Zhou and Lu, 2016; Cheng and Tse, 2018). For example, in pancreas cancer, elevated levels of PIN1 were shown to cooperate with MYC and NRF2 to maintain redox balance, allowing for tumor cell proliferation and survival (Liang et al., 2019). In a mouse model of B-cell lymphoma, loss of PIN1 suppresses MYC-driven proliferation and lymphomagenesis (D’artista et al., 2016). In breast cancer, the overexpression of PIN1 can regulate Notch signaling and increase cancer stem cell-like phenotypes, including tumorigenicity and drug resistance (Luo et al., 2014; Rustighi et al., 2014). PIN1 also enhances the tumorigenic characteristics of mutant p53 in breast cancer by co-activating aggressive oncogenic transcriptional programs. When PIN1 expression is decreased, the malignant activity of mutant p53 is remarkably reduced (Girardini et al., 2011). A more comprehensive list of oncogenes and tumor suppressors that PIN1 can target is reviewed elsewhere (Zhou and Lu, 2016).

Here, we discuss the role of PIN1 as a critical controller of dynamic phosphorylation signaling cascades in response to extrinsic signals that governs gene transcription to alter phenotypic responses in normal and diseased states. PIN1 affects a variety of target transcription factors in such cascades, but we focus on work describing PIN1’s temporal and spatial control of the bHLH-LZ transcription factor c-MYC (hereafter MYC), which PIN1 functionally regulates in both physiologic and pathologic responses. We will describe how PIN1-dependent isomerization temporally and spatially influences the phosphorylation cascade that affects MYC stability and activity in the nucleus. Together, these roles frame PIN1 as a promising therapeutic target for controlling oncogenic MYC.

## PIN1 Regulates Myc Stability and Activity

The proto-oncogene *MYC* encodes a critical transcription factor that influences transcription across the genome to control a multitude of cellular processes including proliferation, survival, metabolism, and morphology (Fernandez et al., 2003; Chen H. et al., 2018). In physiologic conditions, MYC protein levels are mitogen responsive and are influenced by two sequential and interdependent, proline-directed phosphorylation events on Ser62 (pS62) and Thr58 (pT58) in the conserved MYC Box 1 (MB1) region of MYC’s transactivation domain. Phosphorylation at each site influences PIN1’s interaction with the MB1 region of MYC and isomerization at Pro63 (Farrell et al., 2013; Helander et al., 2015). Briefly, MYC is stabilized and activated downstream of growth stimuli through RAS-induced kinases and/or cyclin-dependent kinases (CDKs), which phosphorylate MYC at Ser62 when Pro63 is in *trans* (Sears et al., 2004; Vervoorts et al., 2006). Phosphorylation of Ser62 primes MYC for subsequent phosphorylation at Thr58 by the processive GSK3 kinase (Gregory et al., 2003). Phosphorylation at Thr58 then facilitates the proline-directed, *trans*-specific phosphatase, PP2A-B56α, to remove the activating S62 phosphate (Arnold and Sears, 2006; Arnold et al., 2009). pT58-MYC is then targeted for ubiquitination by the E3 ubiquitin ligase Fbw7, resulting in MYC’s degradation (Gregory and Hann, 2000; Welcker et al., 2004).

**FIGURE 1:**
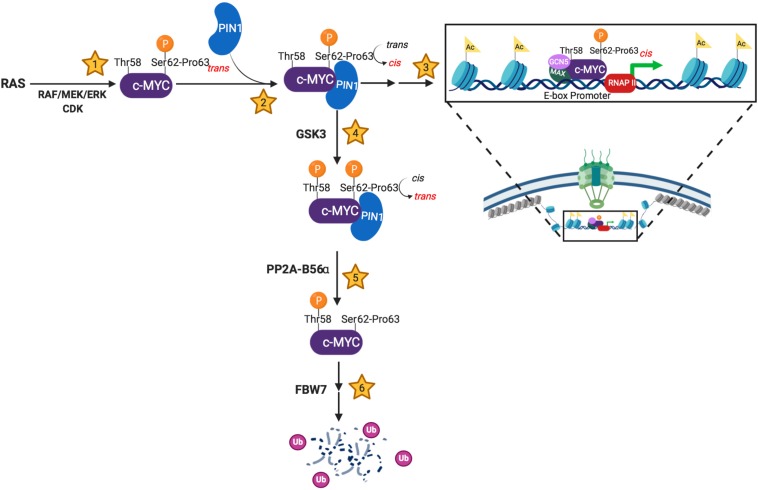
Schema showing PIN1’s involvement in the molecular events regulating MYC’s stability and activity. **(1)** c-MYC becomes transcriptionally active following Ser62 phosphorylation by *trans*-specific RAS-induced kinases and/or cyclin-dependent kinases (CDKs). **(2)** PIN1 stabilizes pSer62-Pro63-MYC in the *cis* conformation, sterically preventing phosphatase activity. **(3)** The transcriptionally active, pSer62-*cis*-Pro63-MYC has increased DNA binding to E-box promoters and increased co-activator association (MAX, GCN5, etc.), which results in increased chromatin accessibility. Additionally, PIN1-directed isomerization of pSer62-MYC has been shown to locate MYC to the basket of the nuclear pore. **(4)** c-MYC is directed towards degradation via Thr58 phosphorylation by the GSK3 kinase. This phosphorylation event promotes phosphatase activity at pSer62, which requires a *cis* to *trans* isomerization of pSer62-Pro63-MYC. **(5)** The *trans*-specific phosphatase, PP2A-B56α, removes the activating phosphate on Ser62-MYC. **(6)** pThr58-MYC signals the E3-ubiquitin ligase, FBW7, to poly-ubiquitinate c-MYC, leading to proteasome degradation. Created with Biorender.com.

As depicted in Figure 1, PIN1 plays a critical role regulating MYC stability and activity, as the kinases and phosphatase that target Ser62 and Thr58 are *trans*-specific enzymes. Thus, PIN1 can interrupt the progression of pS62-MYC through its degradation cascade by stabilizing Pro63 in the *cis*-conformation. This sterically protects the Ser62 phosphate from PP2A-mediated dephosphorylation, allowing for prolonged pS62-MYC interaction with DNA and increasing target gene transcription (Farrell et al., 2013). However, PIN1 can also direct MYC toward degradation following GSK3 phosphorylation of Thr58, associated with subsequent Ser62 dephosphorylation by the *trans*-specific phosphatase, PP2A-B56α (Yeh et al., 2004). Like Ser62, Thr58 is followed by a proline; however, Proline 59 falls within a poly-proline domain, likely structured as a rigid *trans* isomer helix (Andresen et al., 2012). Thus, while Thr58 phosphorylation introduces an additional binding site for PIN1, PIN1-mediated isomerization of MB1 is likely to center on the sterically more flexible Proline 63. From this, we speculate that the re-engagement of PIN1 with pT58 drives a *cis*-*trans* isomerization of Pro63, allowing for the function of PP2A at pSer62. However, additional research is required to understand precisely how Thr58 phosphorylation promotes the dephosphorylation of pSer62, and how this additional phosphorylation affects PIN1’s activity on MYC.

Structural studies into PIN1’s substrate interactions indicate that a flexible interdomain, which connects PIN1’s WW phospho-substrate binding domain to its PPIase catalytic domain, can exist in different rigidity states that influence PIN1 target binding and isomerase activity (Namanja et al., 2011). Furthermore, a study involving molecular dynamic simulations of PIN1 binding suggests that the two subdomains are allosterically regulated in a two-step mechanism. Upon initial substrate binding, PIN1 is primed in an enzymatically quiescent state until the substrate becomes phosphorylated and engages PIN1’s WW domain, triggering PIN1-dependent isomerization (Guo et al., 2015). In support of both primed and activated states for PIN1, a study specifically investigating PIN1’s physical interactions with MYC demonstrated that PIN1 binds to unphosphorylated MYC at a conserved motif, designated MYC Box 0 (MB0), N-terminal to MB1 (Helander et al., 2015). This pre-anchoring of PIN1 to the MB0 region resembles the first quiescent state of PIN1’s substrate engagement, which precedes Ser62 phosphorylation. Phosphorylation of Ser62 triggers PIN1’s WW domain binding and subsequent isomerization of Pro63. However, phosphorylation of Ser62 also increases the dissociation rate of PIN1 from MB1, suggesting release following enzymatic conversion of Pro63 to *cis*. This dynamic interaction may provide a rational role for the additional phosphorylation at Thr58 to re-engage PIN1 with MB1 to mediate a second isomerization event from *cis* to *trans* at the more flexible Pro63. The dual function of PIN1 in promoting both MYC’s activity and degradation through two isomerization events is supported by experiments assessing the effects of point mutations in the MB0 domain that disrupt PIN1 pre-anchoring or of PIN1 knockdown. Both conditions result a reduction in MYC DNA binding and a corresponding decrease in target gene activation, cellular proliferation, and cellular transformation, even though there is an increase in pS62-MYC and MYC stability (Farrell et al., 2013; Helander et al., 2015).

In addition to directly controlling the conformation of MYC to affect its activity vs. ubiquitination, other proteins regulate and are regulated by PIN1 that contribute to the MYC degradation pathway. For example, PIN1 can downregulate the E3 ubiquitin ligase FBW7 (Min et al., 2012), which could disrupt MYC degradation. SENP1 is an enzyme that deSUMOylates MYC, which reduces MYC’s FBW7-directed ubiquitination and degradation; SENP1 also deSUMOylates PIN1 (Chen et al., 2013), which increases PIN1’s activity (Sun et al., 2018). PIN1 is also subject to phosphorylation that can decrease its catalytic activity (Lee et al., 2011). These additional players and levels of post-translational control likely contribute to the differential regulation of PIN1 on MYC in physiologic and pathologic conditions; however, the molecular details require additional research.

## PIN1 Regulates Temporal and Spatial Dynamics of Myc

Understanding the dynamics of MYC regulation is critical in order to elucidate the pleiotropic effects of MYC in the genome and its control of diverse cellular phenotypes. PIN1 plays a key role in this regulation by imparting both temporal and spatial regulation of MYC activity in the nucleus. Temporal studies of MYC DNA binding revealed that MYC oscillates on and off DNA at E-box containing promoters in response to cell growth signaling (Farrell et al., 2013). This dynamic binding of MYC to DNA is dependent on Ser62 and Thr58 phosphorylation and PIN1-mediated Pro63 isomerization. Timed MYC DNA binding assays indicate that phosphorylation of Ser62 accelerates MYC E-box promoter binding in a PIN1-dependent manner while Thr58 phosphorylation accelerates the release of MYC from DNA. This mechanism creates an oscillatory binding of MYC to target gene promoters with a periodicity of approximately 20 min, and loss of PIN1 suppresses this cyclic DNA binding. The temporal control of MYC by PIN1 also regulates its association with its co-activators, which similarly oscillate on and off DNA, in a PIN1-dependent manner, with the same kinetics as MYC (e.g. p300, GCN5, CDK9, and SNF5). MYC’s dynamic binding to coactivators and DNA affects subsequent gene expression by triggering RNA polymerase release and elongation (Jaenicke et al., 2016). Inhibition or reduction in PIN1 levels results in decreased MYC oscillation on DNA and decreased MYC-dependent gene expression, even with an observed increased in MYC protein levels (Farrell et al., 2013).

In addition to temporally regulating MYC activity, PIN1 regulates the subnuclear localization of MYC under normal mitogen stimulation conditions, during wound healing, and in cancer cell lines (Su et al., 2018). Initial observations of MYC at the nuclear periphery were recently extended to show that transcriptionally active pS62-MYC associated with Lamin A/C (Eisenman et al., 1985; Vriz et al., 1992; Myant et al., 2015). This observation is surprising since the majority of chromatin in lamin-associated domains (LADs) at the nuclear periphery is transcriptionally silent heterochromatin. At the nuclear pore (NP), however, there are regions of open chromatin that are poised for transcription (Blobel, 1985; Krull et al., 2010; Beck and Hurt, 2017). Using proximity ligation assay (PLA) with confocal microscopy and super-resolution stochastic optical reconstruction microscopy (STORM), we showed that pS62-MYC associated with the interior basket proteins of the NP complex (NPC) (Su et al., 2018). Although the mechanism of pS62-MYC trafficking to the NP remains unclear, PIN1-mediated isomerization is necessary for stabilizing pS62-MYC at the NPC. In addition, the recruitment of MYC-associated coactivators and epigenetic modifiers, such as GCN5, to the NPC is also PIN1-dependent. This PIN1-dependent spatial reorganization of MYC appears to impact epigenetic regulation in response to extrinsic signals. Upon serum stimulation in starved MEFs, the PIN1-dependent trafficking of pS62-MYC and its associated epigenetic modifiers to the NP results in increased histone acetylation and transcription of NPC-resident genes. Whether this also involves oscillatory DNA binding by MYC at these NPC-resident genes will require future research. Global chromatin accessibility assays indicate that early response chromatin site opening is PIN1-dependent and overlaps with MYC gene program activation, suggesting that these early events involve NPC-associated euchromatin. In the absence of PIN1, the cellular response to mitogen stimulation is delayed, which results in reduced cellular proliferation as well as decreased MYC-associated chromatin remodeling, supporting a critical role for PIN1-MYC regulation of NPC associated euchromatin for efficient response to cellular stimulation.

The PIN1-driven spatial reorganization of MYC to specific chromatin domains at the NP suggests that post-translational control of transcription factors in response to environmental signals may dictate their involvement in regulating specific topologically associated domains or TADs. Interestingly, the number and composition of NPs is increased and altered in cancer cells (Simon and Rout, 2014; Rodriguez-Bravo et al., 2018). In addition, the NP region is speculated to be a site of epigenetic memory for genes associated with rapid response to environmental signals (D’urso and Brickner, 2014). PIN1 drives a relocation of MYC to chromatin regions at the NP, and if these regions comprise a subset of rapid response genes, this could provide a mechanism for MYC’s differential activity on subsets of cell-context specific genes (Sabò et al., 2014; Su et al., 2018).

These findings suggest that in response to extrinsic signals, PIN1 facilitates the generation of a distinct pool of post-translationally modified MYC that associates with chromatin near the inner basket of the NP. This pool may be distinct from the population of MYC within the nuclear interior that binds promoter regions in open chromatin. There is much discussion in the field for whether oncogenic MYC acts as a global transcriptional amplifier or if there is a more specific MYC-driven gene program that drives malignancies (Loven et al., 2012; Nie et al., 2012; Sabò et al., 2014; Caforio et al., 2018; Muhar et al., 2018). Our data suggest that the PIN1-dependent subnuclear reorganization of MYC into distinct pools might allow a population of MYC to drive a specific subset of genes, while the PIN1-independent population may accomplish its global transcriptional amplification function. Future investigation into the dynamic distribution of MYC’s transcriptional activity is necessary for bolstering this hypothesis.

## Conclusion

Here we present a perspective of the role of PIN1 in regulating dynamic response phenotypes, focusing on its isomerization of MYC in multiple cellular contexts. PIN1’s interaction with and isomerization of MYC supports the physiologic and oncogenic activity of MYC (Yeh et al., 2004; Farrell et al., 2013; Sanchez-Arévalo Lobo et al., 2013; Helander et al., 2015; Su et al., 2018). Mechanistically, this involves regulation of MYC stability, its DNA binding and transcriptional activity, and its subnuclear localization to the NP. In normal cells, PIN1’s regulation of MYC contributes to increased proliferation, migration, and wound healing (Su et al., 2018). In cancer, PIN1’s regulation of MYC has been shown to affect oncogenic transformation, proliferation, redox maintenance, and cell survival (Farrell et al., 2013; Helander et al., 2015; D’artista et al., 2016; Su et al., 2018; Liang et al., 2019). PIN1 fine-tunes the rapid spatial and temporal control of MYC by integrating isomerization of Pro63 with the sequential phosphorylation events at Ser62 and Thr58 (Figure 1). Whether the dynamic nature of PIN1-dependent regulation of MYC extends to PIN1-dependent regulation of other transcription factors will be of great interest.

Multiple efforts to therapeutically reduce or control MYC’s oncogenic activity have been unsuccessful for several reasons, including an inability to specifically control MYC expression and the lack of an enzymatic region to target with small molecules (Chen H. et al., 2018). The direct targeting of PIN1 to modulate MYC activity provides a promising therapeutic opportunity with numerous drugs under investigation (Chen Y. et al., 2018). For example, the inhibition of PIN1 with PiB reduced the rate of MYC binding to target DNA promoters in MCF10A cells, leading to decreased expression of oncogenic gene signatures and decreased tumor growth (Farrell et al., 2013). In addition, Juglone (Kim et al., 2009) and ATRA (Wei et al., 2015) have been shown to potently reduce PIN1’s oncogenic activity in breast cancer models; however, the efficacy of these drugs on reducing MYC’s oncogenic activity remains to be studied. Furthermore, a recent covalent PIN1 inhibitor, KPT-6566, has shown potency for reducing PIN1-dependent cancer phenotypes (Campaner et al., 2017). Since PIN1 null mice are viable, taking advantage of the upstream functional control of phosphorylated MYC via PIN1 enzymatic blockade could reduce systemic toxicity associated with total loss of MYC, while specifically targeting signaling-activated oncogenic MYC. This specificity provides a compelling rationale for PIN1-dependent therapeutic strategies to treat MYC-dependent cancers.

## Author Contributions

GC contributed to writing all sections of the manuscript. DL and EL wrote sections of the manuscript. EL organized and oversaw the conceptual approaches. RS oversaw conceptual approaches, edited all sections of the manuscript, and approved the manuscript. All authors contributed to manuscript revision, read, and approved the submitted versions.

## Conflict of Interest

The authors declare that the research was conducted in the absence of any commercial or financial relationships that could be construed as a potential conflict of interest.
